# Emerging roles of traditional Chinese medicine in the treatment of diabetic gastroparesis

**DOI:** 10.3389/fnut.2025.1680943

**Published:** 2025-11-17

**Authors:** Hong Zhang, Qianhui You, Xiaohong Feng, Zhengran Qin

**Affiliations:** 1 Department of Endocrinology and Metabolism, The First Affiliated Hospital of Zhejiang Chinese Medical University, Hangzhou, China; 2 Department of Anatomy, School of Integrative Medicine, Shanghai University of Traditional Chinese Medicine, Shanghai, China

**Keywords:** traditional Chinese medicine, diabetic gastroparesis, therapeutic agent, interstitial cells of Cajal, oxidative stress, gut microbiota homeostasis

## Abstract

Diabetic gastroparesis (DGP), a prevalent manifestation of diabetic autonomic neuropathy, is characterized by impaired gastrointestinal motility and delayed emptying. The pathogenesis of DGP is multifactorial, with chronic hyperglycemia serving as a key contributor through its induction of oxidative stress, inflammatory responses, immune dysregulation, and gut microbiota imbalance. These pathological processes collectively induce injury to interstitial cells of Cajal (ICCs) and gastric smooth muscle cells (GSMCs), accompanied by neuromuscular dysfunction, ultimately resulting in gastroparesis and its associated clinical symptoms. In the management of DGP, traditional Chinese medicine (TCM) offers distinct advantages, including demonstrated efficacy, favorable safety profiles, personalized treatment approaches, excellent patient tolerance, diverse dosage forms, and multi-target therapeutic effects. While the precise mechanisms remain to be fully elucidated, emerging evidence suggests that TCM may exert its beneficial effects through the protection of ICCs and GSMCs, modulation of brain-gut peptide dysregulation and neuropathy, regulation of inflammatory and immune responses, attenuation of oxidative stress, and restoration of gut microbiota homeostasis. Notably, current evidence supporting TCM for DGP leans on preclinical data with a focus on positive outcomes, whereas clinical evidence is sparse and largely limited to Chinese cohorts, reflecting the need for more clinical data and multi-cohort studies. This review comprehensively summarizes recent advances in understanding the mechanisms underlying TCM-mediated prevention and treatment of DGP, intending to provide a scientific foundation for expanding the clinical application of TCM in DGP management.

## 1 Introduction

Diabetic gastroparesis (DGP) is a common autonomic neuropathy of diabetes, characterized by delayed gastric emptying (GE) in the absence of mechanical outlet obstruction and other etiologies that may delay GE. Its main clinical symptoms include early satiety, nausea, vomiting, bloating, abdominal pain, diarrhea, and constipation, which further exacerbate impaired glucose regulation and malnutrition, thereby significantly affecting patients’ quality of life (1). Bharucha et al. (2) review indicated that up to 50% of patients with moderately controlled type 1 and type 2 diabetes mellitus (DM) have GE. The exact pathogenesis of DGP has not been fully elucidated, but current research suggests its pathological processes involve multiple factors: structural and functional damage to interstitial cells of Cajal (ICCs) and gastric smooth muscle cells (GSMCs); abnormal secretion of brain-gut peptides and neuropathy; inflammatory responses and immune dysregulation; oxidative stress; and gut microbiota imbalance. It is worth noting that these pathogenic factors are not only closely associated with chronic hyperglycemia but also interact with each other to jointly promote the occurrence and progression of DGP (3, 4).

Based on the above pathogenesis, common treatment methods for DGP in Western medicine include gastroprokinetic agents, antiemetics, antacids, antibiotics, gastric electrical stimulation, and new surgical treatments represented by gastric per-oral endoscopic myotomy (2, 5). However, these methods may lead to drug resistance, poor efficacy, and other adverse effects such as arrhythmia, hyperprolactinemia, bleeding and ulceration, which limit their clinical application in patients with DGP (2, 6). Therefore, there is a need to identify safer and more effective therapeutic strategies to treat DGP.

Traditional Chinese Medicine (TCM) has a 2000-year-long history in managing gastrointestinal disorders, such as refractory nausea, vomiting, and abdominal distension. According to TCM theory, DGP can be classified into different categories based on its clinical symptoms, such as “Xiao Ke disease” (wasting-thirst disease) combined with “Pi Man” (abdominal distension), “Wei Tong” (epigastric pain) and “Ou Tu” (vomiting). The fundamental pathogenesis of DGP resides in the spleen-stomach dysfunction, defined by imparied transportation and transformation, which induces phlegm turbidity, blood stasis, and “Qi” (the vital energy that circulates throughout the body) stagnation (7). Based on this theoretical framework, modern Chinese medicine scholars have proposed new perspectives. For instance, Professor Cui Shusheng suggests that the primary disease locations in DGP are the spleen, stomach, and intestines, with significant connections to the liver, lungs, and kidneys. In the early stage, disharmony in the descending function of the lungs and stomach leads to fluid injury and dryness-heat, warranting treatment by regulating the lungs and stomach simultaneously to moisten dryness and unblock the intestines. In the intermediate stage, impaired liver Qi flow triggers upward stomach Qi counterflow, which is treated by soothing the liver, regulating the spleen, harmonizing the stomach, and guiding the counterflow downwards. In the late stage, there is a dual deficiency of “Qi” and “Yin” (the nourishing, cooling, and grounding aspect of the body that counters the active, warm, and outward energy of “Yang”), coexisting with blood stasis and turbidity. At this stage, treatment focuses on tonifying “Qi,” nourishing “Yin,” eliminating stasis, and resolving turbidity (8). Professor Tong Xiaolin argues that DGP can be divided into an acute phase and a remission phase. The acute phase primarily involves the inversion of stomach “Qi,” “Yang” (the active, warm, and stimulating energy that drives bodily processes) deficiency in the spleen and stomach, with the main therapy focus on relieving symptoms such as vomiting and stomach distention. Commonly used TCM herbs include Xiaobanxia Decoction, Zhizhu Decoction. During the remission phase, the most common TCM syndrome patterns include cold deficiency in the “Middle Jiao” (the middle region of the body associated with the spleen and stomach, responsible for digestion, absorption, and the transformation of food into energy and nutrients to sustain bodily nourishment and vitality), and spleen-kidney “Yang” decline. The treatment principles focus on warming “Yang” to dissipate cold and boosting “Qi” to strengthen the spleen, with representative prescriptions such as Fuzi Lizhong Decoction and Xuanfu Daizhe Decoction (9). Beyond the oral administration of TCM herbal formulations, acupuncture and moxibustion exert notable bidirectional regulatory effects in gastrointestinal disorders, effectively restoring balance to both hyperactive and weakened digestive functions. Zusanli (ST-36), a primary acupoint for gastric disorders, regulates spleen-stomach function and promotes gastrointestinal motility. Sanyinjiao (SP-6), where the three “Yin” meridians converge, strengthens the spleen, harmonizes the stomach, and nourishes the “Yin” to moisten the intestines. Liangmen (ST-21), adjacent to the stomach, can relieve gastric reflux and dissolve food stagnation. Combined acupuncture of Zusanli, Liangmen, Sanyinjiao acupoints and so on, synergistically enhances spleen-stomach harmony and promotes “Qi” circulation to relieve stagnation (10).

In recent years, accumulating evidence has demonstrated that based on the theory of syndrome differentiation and treatment in TCM, the application of TCM therapies, including oral herbal formulations, external acupuncture and moxibustion, or a combination of internal and external treatments, can effectively alleviate the key symptoms of DGP, such as nausea, vomiting, abdominal distension, and pain. Notably, TCM interventions are characterized by minimal invasiveness, high safety, and low recurrence rates, underscoring their significant potential in DGP management (11–14). However, from a pathophysiological perspective, the mechanisms underlying the therapeutic efficacy of TCM in DGP remain incompletely elucidated, which has limited its evidence-based validation and broader clinical adoption. To the best of our knowledge, this review represents the first comprehensive analysis to systematically elucidate the multifaceted mechanisms of TCM in DGP treatment, including the protection of ICCs and GSMCs, and the modulation of brain-gut peptides dysregulation and neuropathy, the regulation of inflammatory and immune responses, inhibition of oxidative stress, and improvement of gut microbiota imbalance. This work not only deepens our understanding of the therapeutic potential of TCM in treating DGP but also provides a scientific basis for further clinical application. By integrating TCM theories with modern biomedical research, this review bridges the gap between empirical knowledge and scientific validation, offering a holistic perspective on the therapeutic mechanisms of TCM in treating DGP.

## 2 Mechanisms of TCM in treating DGP

TCM ameliorates diabetic gastroparesis through multiple mechanisms: alleviating interstitial cells of Cajal (ICCs) injury, regulating brain-gut peptides and neuropathy, attenuating gastric smooth muscle cells (GSMCs) damage, modulating inflammation/immunity, inhibiting oxidative stress, and restoring gut microbiota homeostasis, thereby improving gastrointestinal motility (Table 1).

**Table 1:** Traditional Chinese medicine in the treatment of diabetic gastroparesis.

TCM therapy	Experimental model	Effects	Mechanism
Banxia Xiexin Decoction (BXD) (22)	*In vivo*: db/db mice with DGP model	Reduce the gastric half-emptying time	Promote ICCs proliferation; Inhibiting the expression of AGEs and RAGE; Promoting nNOS expression
Banxia Xiexin Decoction (BXD) (73, 96)	*In vivo*: SD rats with DGP model	Repair the pathological injury of the intestinal mucosa; Reduce the gastric residual rate; Improve intestinal propulsive rate	Inhibit inflammatory responses; Regulate intestinal mucosal immune responses; Alleviate damage to the intestinal mucosal barrier
Chaiqin Wendan Decoction (CWD) (50)	DGP patients	Shorten the gastric emptying time; Increase the frequency, amplitude and rhythm of gastric electricity; Improve clinical symptoms	Regulate the brain-gut peptideslevels
FoxiangSan (98)	*In vivo*: SD rats with DGP model	Decrease the gastric residue levels	Modulate gut microbiota
Jinqi Zhizhu Decoction (JZD) (48, 49)	*In vivo*: GK rats with DGP model	Reduce the gastric residual rate and improve gastric motility	Promote neurotransmitter secretion; Increase the gastric smooth muscle contraction and repair damage of GSMCs by upregulating the RhoA/ROCK pathway
Atractylenolide-1 (1)	*In vivo*: SD rats with DGP model	Enhance the frequency of gastric blood flow; Increase the amplitude of gastric peristalsis; Restore the gastric emptying rate	Reduce ICCs’ apoptosis and suppress oxidative stress
*Alpinia officinarum* Hance (AOH) (97)	*In vivo*: SD rats with DGP model	Increase the gastric emptying and small intestinal propulsive rate	Rebalance gut microbiota
*Alpinia officinarum* Hance – *P. cablin* (Blanco) Benth drugpair (AP) (86)	*In vivo*: C57BL/KsJ db/db and db/m mice with DGP model *In vitro*: GSMCs	Increase the gastric emptying rate	Inhibit the GSMCs’ apoptosis by decreasing oxidative stress levels
Berberine (47)	*In vivo*: SD rats with diabetes and disorders gastric fundus contractile *In vitro*: gastric fundus tissue	Enhance the contractile response of the gastric fundus smooth muscle	Improve the neurological dysfunction of the gastric fundus by promoting the release of Ach in intermuscular plexus of the gastric fundus smooth muscle through the opening of calcium ion channels
Curcumin (87)	*In vivo*: smooth muscle of the gastric antrum of DGP rats	Improve the gastric emptying rate	Inhibit the ICCs’ apoptosis by reducing oxidative stress levels through abolishing the NF-κB signal transduction and enhancing the expression of SCF/c-kit
Curcumin/Cinnamaldehyde (88)	*In vivo*: Nrf2 KO and WT mice with DGP model	Improve delayed gastric emptying	Inhibit inflammation and oxidative stress; Attenuate impairment of gastric nitrergic relaxation
Ethyl acetate Extract of *Salsola collina* (EES) (23)	*In vivo*: SD rats with DGP model	Improve the gastric emptying rate	Improve the quantity and function of ICCs by upregulating the c-Kit/SCF signalling; Regulate brain-gut peptides excretion
Emodin (31)	*In vivo*: SD rats with DGP model	Increase the gastrointestinal transmission rate	Inhibit the colon over-autophagy of ICCs; Regulate intestinal motility-associated neurotransmitters
Hedysari radix polysaccharide(HRP) (59)	*In vivo*: Wistar rats with DGP model	Increase the gastric emptying rate	Protect the GSMCs against apoptotic injury by activating the IGF-1/PI3K/Akt signalling
Hedysari radix polysaccharide(HRP) (60)	*In vivo*: Wistar rats with DGP model	Increase the gastric emptying rate	Inhibit the pyroptosis of GSMCs
Hedysari radix polysaccharide(HRP) (85)	*In vivo*: Wistar rats with DGP model	Improve the gastric emptying rate and intestinal propulsion rate	Inhibit oxidative stress and repair small intestinal mucosal damage
Higenamine (62)	*In vivo*: SD rats with DGP model	Repair the weakened gastric emptying ability	Promote the GSMCs’ proliferation and inhibit the GCMSs’ apoptosis by activating the β-AR/PI3K/AKT signalling
Huanglian-Banxia herb pair (HL-BX) (39)	*In vivo*: SD rats with DGP model	Improve the gastric emptying rate and intestinal propulsion rate	Modulate the brain-gut neurotransmitters
Electroacupuncture at Zusanli, Sanyinjiao, and Liangmen acupoints (EA) (17)	*In vivo*: SD rats with DGP model	Improve the gastric emptying rate	Promote the autophagy of ICCs and reduce the apoptosis of ICCs
Electroacupuncture at Zusanli, Sanyinjiao, and Liangmen acupoints (EA) (33)	*In vivo*: SD rats with DGP model	Improve the gastric emptying rate	Promote autophagy of ICCs through down-regulating the PI3K/Akt/mTOR signalling
Electroacupuncture at Zusanli acupoint (EA) (51)	*In vivo*: SD rats with DGP model	Increase the gastric emptying rate, the mean antral pressure, and the amplitude of antral motility	Reduce the loss of neurotransmitters and neurons in the ENS
Electroacupuncture at Zusanli, Sanyinjiao, and Liangmen acupoints (EA) (52)	*In vivo*: SD rats with DGP model	Improve the gastric emptying rate and intestinal propulsion rate	Regulate diabetic neuropathy
Electroacupuncture at Zusanli, Sanyinjiao, and Liangmen acupoints (EA) (53)	*In vivo*: SD rats with DGP model	Improve the gastric emptying rate and intestinal propulsion rate	Regulate the brain-gut peptides levels
Electroacupuncture at Zusanli, Sanyinjiao, and Liangmen acupoints (EA) (78)	*In vivo*: SD rats with DGP model	Increase the gastric emptying and small intestinal propulsive rate	Inhibit inflammatory factors
Electroacupuncture at Zusanli, Sanyinjiao, and Liangmen acupoints (EA) (79)	*In vivo*: SD rats with DGP model	Repair the pathological damage in the gastric antrum tissues; Increase the gastric emptying and small intestinal propulsive rate	Inhibit pyroptosis in the gastric antrum tissues by attenuating the NLRP3/caspase-1/GSDMD pathway
Electroacupuncture at Zhongwan, Tianshu, Zhongfu, bilateral Zhangmen, Taibai, Taiyuan, Zusanli, and Shangjuxu acupoints (EA) (102)	DGP patients	Improve the symptoms of DGP patients (GSRS and GCSI scores)	Affect the distribution of gut microbiota
Point-moxibustion at Zusanli, Sanyinjiao, and Liangmen acupoints (52)	*In vivo*: SD rats with DGP model	Improve the gastric emptying rate and intestinal propulsion rate	Regulate diabetic neuropathy
Low-intensity pulsed ultrasound stimulation at acupoint ST36 (LIPUS) (63)	*In vivo*: SD rats with DGP model *In vitro*: circular musclestrips excised from the gastric antral specimen of the rat	Improve gastric emptying	Improve the GSMCs’ contractile ability by upregulating the RhoA/Rock pathway and modulating the MALAT1/miR-449a/DLL1 pathway
Oral use of modified Sini Hewei Anshen Decoction (SHAD) combination with the navel application of Jianpi Hewei Adhesive Plaster (76)	DGP patients accompanied by anxiety due to liver-stomach disharmony	Improve GCSI and TCM symptom scores; Shorten gastric emptying time	Inhibit inflammatory factors
Oral use of Chaihu Shugan San (CSS) combination with acupuncture (77)	DGP patients	Improve TCM symptom scores	Inhibit inflammatory factors
Electroacupuncture at Zhongwan, Neiguan, and Sanyinjiao acupoints combination with “Zhuang” medicine thread moxibustion (89)	*In vivo*: SD rats with DGP model	Improve the gastric emptying rate and intestinal propulsion rate	Inhibit oxidative stress-induced damage to gastric smooth muscle tissue
Acupuncture in combination with oral use of domperidone (90)	DGP patients diagnosed with a liver stagnation and spleen deficiency pattern	Improve clinical symptoms (decrease the TCM symptom and GCSI scores); Improve the gastric emptying rate	Inhibit the oxidative stress and inflammatory responses

### 2.1 Reducing ICCs injury

The interstitial cells of Cajal (ICCs) are specialized mesenchymal cells located throughout the gastrointestinal tract. As pacemaker cells, ICCs are responsible for generating and propagating slow-wave electrical potentials, which are critical for coordinating the rhythmic contractions of GI smooth muscle (15). ICCs also serve as critical intermediaries between enteric neurons and smooth muscle cells (SMCs). They facilitate the transmission of both excitatory and inhibitory signals that govern smooth muscle activity (16). The impairment or loss of ICCs disrupts antral peristaltic function, resulting in diminished gastric motility and subsequent delays in gastric emptying (17). Studies have also identified a significant decrease in the number of ICCs and ultrastructural alterations, including outer membrane rupture, intracellular mitochondria shrinkage, membrane wrinkling, cristae disappearance in the gastric tissue of rats with DGP (18, 19). Moreover, studies have demonstrated a significant loss of ICCs in DGP patients compared to controls (20). These ICCs impairments were observed in the stomach, jejunum, and colon of individuals with diabetic gastroenteropathy, irrespective of diabetes type (type 1 or type 2) (21). Therefore, reducing ICCs injury can be an important target for preventing and treating DGP.

**Figure 1:**
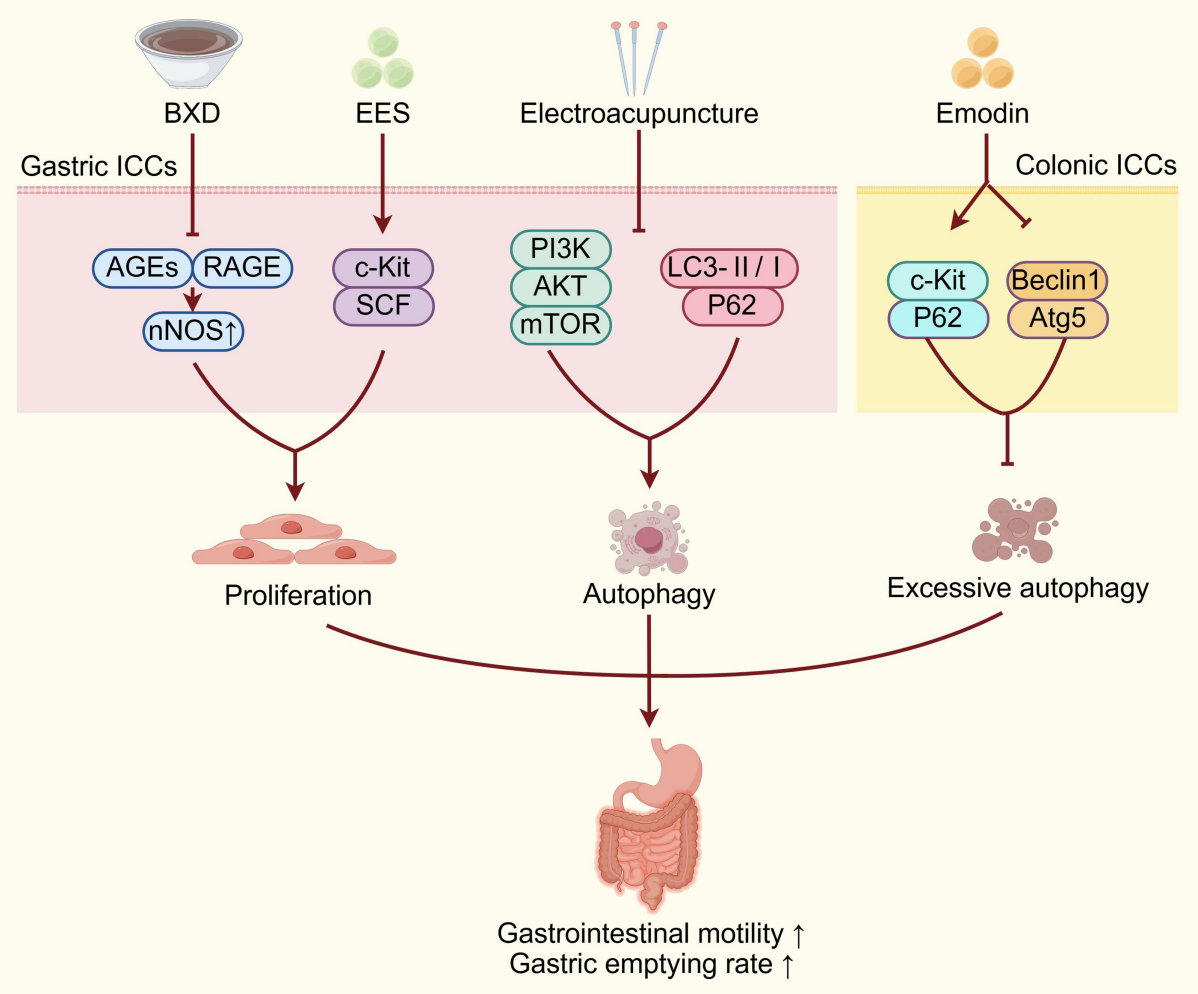
TCM improves DGP by reducing ICCs injury. TCM interventions act on gastric/colonic ICCs via diverse pathways (AGEs/RAGE, c-Kit/SCF, PI3K/Akt/mTOR, autophagy-related proteins) to regulate proliferation, autophagy, and inhibit excessive autophagy, improving DGP by alleviating ICCs injury. BXD, Banxia Xiexin Decoction; AGEs, advanced glycation end products; RAGE, receptor for advanced glycation end products; c-Kit, tyrosine-protein kinase kit; SCF, stem cell factor; ICCs, interstitial cells of Cajal.

It has been confirmed that TCM interventions can effectively improve DGP by decreasing ICCs injury (Figure 1). Banxia Xiexin Decoction (BXD) is a classical prescription of traditional Chinese medicine that is composed of seven key herbs: *Pinellia ternate* (Thunb.) Makino (Banxia), *Scutellaria baicalensis* Georgi (Huangqin), *Zingiber officinale* Roscoe (Shengjiang), *Panax ginseng* C. A. Mey. (Rensheng), *Coptis chinensis* Franch. (Huanglian), *Ziziphus jujuba* Mill. (Dazao), and *Glycyrrhiza uralensis* Fisch (Gancao). Research has demonstrated that BXD can improve gastric motility in DGP mice by inhibiting the expression of advanced glycation end products (AGEs) and advanced glycation product receptors (RAGE), while promoting neuronal nitric oxide synthase (nNOS) expression to regulate ICCs proliferation (22). Additionally, research has also found that the ethyl acetate extract of the TCM herb *Salsola collina* (EES) can significantly enhance gastric emptying in DGP rats. This effect is mediated by improving the number and function of ICCs through the upregulating the c-Kit/SCF signaling pathway (23). The tyrosine-protein kinase kit (c-Kit) serves as a critical regulator of ICCs, mediating both their development and functional integration within the gastrointestinal tract. Through its activation by stem cell factor (SCF), c-Kit facilitates ICC formation and promotes the establishment of functional gap junctions between ICCs and SMCs, thereby maintaining coordinated visceral motility (24–26). Autophagy is a highly conserved, lysosome-dependent degradation pathway that transports intracellular proteins, damaged organelles, and other cellular components to lysosomes for breakdown and recycling, playing a critical role in preserving normal cellular function and ensuring a stable intracellular environment essential for cell survival (17). Excessive autophagy has been implicated in the pathogenesis of ICCs damage and dysfunction, contributing to diabetic gastroenteropathy and intestinal dysmotility under chronic hyperglycemic conditions (27). The level of P62 typically shows an inverse correlation with autophagic flux, as it is degraded during active autophagy (28). Atg5 has a critical function in autophagosome-lysosome fusion, while Beclin1 expression is widely regarded as a key biomarker of autophagy activation; therefore, Atg5 and Beclin1 proteins are upregulated during autophagy activity (29, 30). Research has shown that emodin, as the active component of the TCM herb *Rheum palmatum* L. (Dahuang), can upregulate the expression of c-Kit and P62 proteins, downregulate the expression of autophagy-related proteins Beclin1 and Atg5, thereby inhibiting excessive autophagy of colonic ICCs and improving colonic motility and intestinal defecation disorders in DGP rats (31).

However, autophagy exerts a complex and multifaceted role in the pathogenesis of gastrointestinal motility disorders, as both excessive and insufficient autophagy can act as key contributors to the disease (32). Zhang et al. (33) found that electroacupuncture (EA) at Zusanli, Sanyinjiao, and Liangmen acupoints can promote ICCs autophagy through downregulating the PI3K/Akt/mTOR signaling pathway, thereby improving gastric emptying. The specific mechanism is that EA can suppress the type I PI3K expression, thereby inhibiting phosphatidylinositol (3,4,5)- trisphosphate (PIP3) generation, Akt phosphorylation, and mTOR activation. Through this pathway, EA ultimately enhances autophagy-related gene expression and downstream substrate activity via inhibition of the mTOR-ubiquitin-proteasome pathway to promote cellular autophagy (33). Additionally, electroacupuncture at acupoints such as Zusanli, Sanyinjiao, and Liangmen has also been shown to enhance the autophagy of ICCs and reduce their apoptosis rate. This is evidenced by a decrease in the autophagic flux blockage marker—microtubule-associated protein 1A/1B-light chain 3 (LC3)-II/I ratio, and a reduction in the expression of P62 protein, thereby improving gastric motility in DGP rats (17). Apoptosis, a form of programmed cell death, is critical for regulating cell proliferation and tissue homeostasis. However, the precise mechanism linking autophagy and apoptosis in ICCs remains elusive. In conclusion, TCM interventions such as BXD, EES, emodin and electroacupuncture can ameliorate ICCs injury by regulating proliferation, autophagy and apoptosis, thereby exerting therapeutic effects on DGP.

However, most current studies on these TCM interventions for ICCs protection in DGP are limited by small sample sizes, lack of blinding, and potential bias. The possible reason may lies in standardizing TCM formulations and designing blinded trials for acupuncture, highlighting the need for larger-scale randomized controlled trials (RCTs) in future research.

### 2.2 Modulation of brain-gut peptides dysregulation and neuropathy

Brain-gut peptides constitute a class of highly bioactive molecules that are widely distributed across both the gastrointestinal tract and nervous system, serving as critical regulators of gastrointestinal motility through both excitatory and inhibitory mechanisms (34). Among these regulatory factors, ghrelin and gastrin (GAS) function as potent stimulators of gastrointestinal activity. They enhance digestive juice secretion and promoting smooth muscle contraction to facilitate the propulsion of gastric and intestinal conten while augmenting overall motility (35). Similarly, substance P (SP) and acetylcholine (ACh) act as excitatory neurotransmitters. They significantly contribute to gastrointestinal smooth muscle contraction, intestinal peristalsis, and luminal propulsion (36, 37). Conversely, inhibitory mediators such as somatostatin (SS) and cholecystokinin (CCK) suppress exocrine pancreatic secretion and bile production, consequently attenuating smooth muscle contractility and delaying gastrointestinal emptying (38). Vasoactive intestinal peptide (VIP) serves as an important inhibitory neurotransmitter that modulates gastrointestinal motility by affecting normal intestinal contraction and spontaneous pyloric contraction (23). Additional neurotransmitters, including 5-hydroxytryptamine (5-HT), dopamine (DA), and norepinephrine (NE), further contribute to motility regulation through complex interactions with enteric neurons and direct effects on gastric smooth muscle (39).

The sophisticated regulation of brain-gut peptide secretion and function is mediated by integrated neural networks encompassing the enteric nervous system (ENS), autonomic nervous system, and central nervous system, which operates through multi-layered control mechanisms (13, 40). In the context of diabetes mellitus, chronic hyperglycemia and its associated pathological consequences, including oxidative stress, systemic inflammation, tissue ischemia, and AGE accumulation, collectively contribute to the development of diabetic neuropathy. This manifests through multiple mechanisms: enteric plexus neuronal apoptosis, nerve demyelination, axonal degeneration, disruption of neuronal calcium homeostasis, loss of NOS-containing neurons, and impaired synaptic transmission between extrinsic autonomic neurons and SMCs (41–43). Clinical and experimental evidence supports the association between delayed gastric emptying and neuronal degeneration. This relationship is exemplified in ENS ablation murine models, which exhibit hypoganglionosis, a marked reduction of nNOS-expressing neurons in the gastric antrum, and delayed gastric emptying (44). These findings are further corroborated by clinical studies in human patients with DGP, where full-thickness gastric biopsies reveal significant reductions in circular muscle enteric nerve fibers (45). Mechanistic studies conducted in DGP murine models have revealed that activation of transient receptor potential vanilloid type 1 (TRPV1) channels in gastrointestinal vagal afferent fibers can stimulate nitric oxide (NO) release through modulation of nNOS, resulting in fundic relaxation and consequent improvement of delayed gastric emptying (46). Collectively, these findings highlight the fundamental role of brain-gut peptide dysregulation and neuropathic changes in the pathogenesis of DGP.

**Figure 2:**
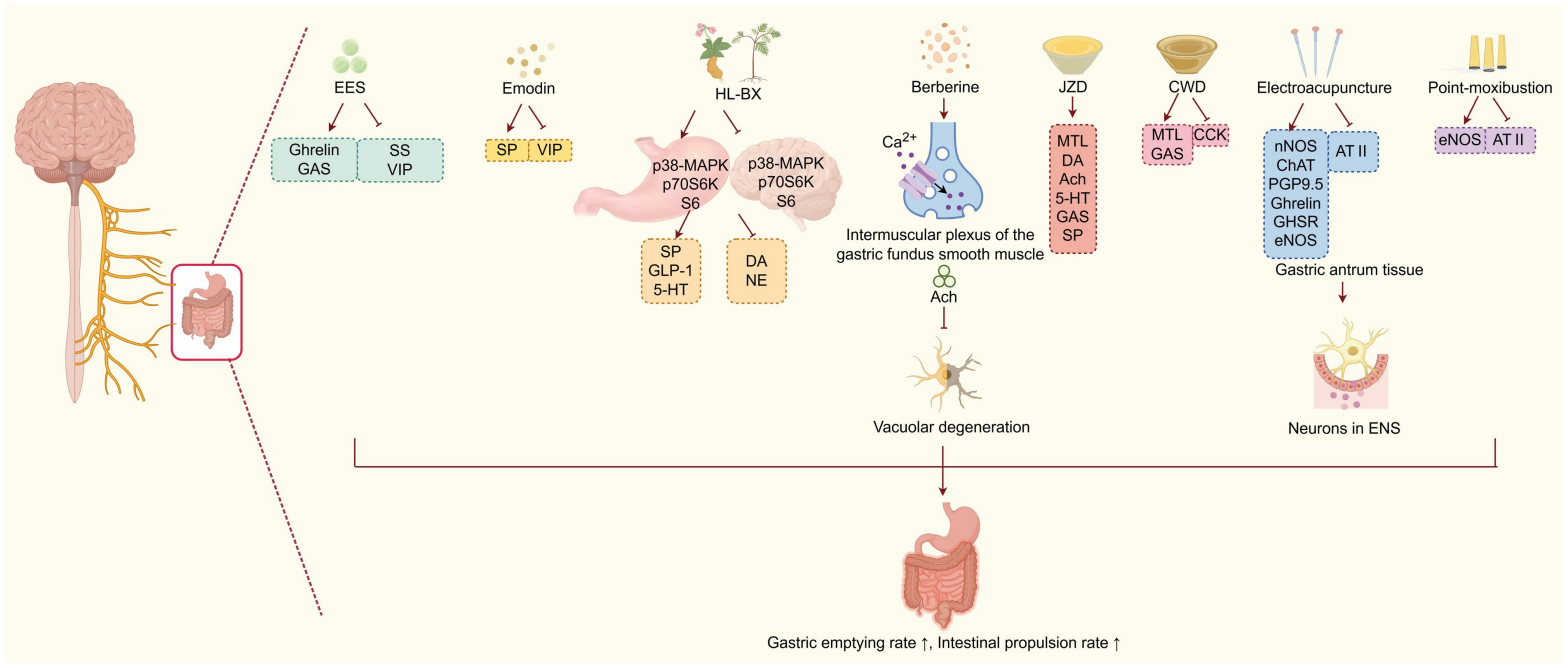
TCM improves DGP by modulating brain-gut peptides and neuropathy. Various TCM interventions (herbs, acupuncture, moxibustion) regulate brain-gut peptides and neural pathways, which suppress vacuolar degeneration and reduce the loss of neurons in the enteric nervous system (ENS), ultimately enhancing gastric emptying and intestinal propulsion.

TCM can effectively improve DGP by modulating brain-gut peptides and neuropathy (Figure 2). For instance, the ethyl acetate extract of *Salsola collina* (EES) can significantly improve gastric emptying in DGP rats. Its mechanism is not only associated with regulating the number and function of ICCs, which serve as the mediator of neuromuscular transmission in the gastrointestinal tract, but also with modulating of brain-gut peptides levels, such as increasing serum ghrelin and GAS levels, while inhibiting the secretion of SS and VIP (23). Similarly, studies have shown that emodin enhances gastrointestinal motility by reducing excessive autophagy in ICCs and increasing serum SP while suppressing VIP concentrations (31). The combined employment of classic TCM herbs *Coptis chinensis Franch.* (Huanglian) and *Pinellia ternate (Thunb.) Makino* (Banxia) has specific effects on nerotransmitters. It increase the levels of SP, glucagon like peptide-1 (GLP-1), and 5-HT, while decreasing DA and NE levels. Further mechanism exploration has shown that the TCM intervention leads to opposing regulation of the MAPK/p70S6K/S6 signalling pathway in both the stomach and brain, thereby promoting gastric motility (39). Berberine, the main active ingredient of the TCM herb Huanglian, has been shown to promote the release of Ach in the intermuscular plexus of the gastric fundus smooth muscle by acting on calcium channels. This action helps improve gastric fundus contraction disorders and alleviate vacuolar lesions in the neuronal cells of the gastric fundus muscle plexus, thereby promoting gastric emptying in DGP rats (47). Moreover, Wan et al. (48, 49)have discovered that Jinqi Zhizhu Decoction (JZD), which consists of TCM herbs such as *Hedysarum polybotrys* Hand-Mazz. (Hongqi), *Citrus aurantium* L. (Zhike), *Atractylodes macrocephala* Koidz. (Baizhu), *Coptis chinensis* Franch. (Huanglian), *Pinellia ternate* (Thunb.) Makino (Banxia), and *Endothelium Corneum Gigeriae Galli* (Jineijin), could effectively reduce the gastric residual rate in DGP rats. Mechanism studies have discovered that he formula can increase serum MTL, plasma DA, ACh, 5-HT, GAS and SP levels. Furthermore, Chaiqin Wendan Decoction (CWD), a traditional Chinese herbal formulation composed of *Bupleurum chinense* DC. (Chaihu), *Citrus reticulata* Blanco. (Chenpi), *Citrus aurantium* L. (Zhishi), *Ziziphus jujuba* Mill. (Dazao), *Bambusa tuldoides* Munro. (Zhuru), *Poria cocos* (Schw.) Wolf (Fuling), *Atractylodes macrocephala* Koidz. (Baizhu), *Paeonia lactiflora* Pall. (Baishao), *Salvia miltiorrhiza* Bunge. (Danshen), *Scutellaria baicalensis* Georgi. (Huangqin), *Pinellia ternate* (Thunb.) Makino (Banxia), and *Glycyrrhiza uralensis* Fisch. ex-DC. (Gancao). This formula has shown therapeutic efficacy in DGP patients. Mechanistically, the formula can enhance gastric motility through increasing the serum MTL and GAS levels, while decreasing the CCK levels (50). Electroacupuncture has also demonstrated its efficacy in treating DGP by modulating brain-gut peptides dysregulation. For instance, Han Xu’s study revealed that electroacupuncture stimulation at the Zusanli acupoint (ST36) can upregulate key neurotransmitters, including nNOS, ChAT, and protein gene product 9.5 (PGP9.5) in gastric antrum tissues. This, in turn, helps reduce the loss of neurotransmitters and neurons in the ENS, significantly improving gastric motility in DGP rats (51). Both point moxibustion and electroacupuncture at the Zusanli, Sanyinjiao, and Liangmen acupoints can enhance the gastric emptying rate and intestinal propulsion in DGP rats. This effect is achieved by increasing the expression of eNOS mRNA and decreasing the expression of Angiotensin II (ATII) mRNA in the gastric antrum. Under pathological conditions, ATII can promote endothelial cells to secrete vasoconstrictor peptides and reduce eNOS activity, leading to gastrointestinal endothelial dysfunction and motility disorders (52). Additionally, electroacupuncture at the Zusanli, Sanyinjiao, and Liangmen acupoints can enhance gastric emptying by increasing the expression of ghrelin and GHSR mRNA in the gastric antrum (53). In summary, TCM treatment including Banxia Xiexin decoction, Jinqi Zhizhu Decoction, Chaiqin Wendan Decoction, the Huanglian-Banxia drug pair, EES, berberine, as well as point moxibustion and electroacupuncture, can help alleviate DGP by modulating brain-gut peptides and neuropathy.

Notably, existing studies in this area often suffer from small sample sizes and insufficient blinding, which may introduce bias. This is partly due to the complexity of measuring brain-gut peptide levels across diverse cohorts, and future research should prioritize well-powered, blinded RCTs to validate these findings.

### 2.3 Attenuating GSMCs damage

Gastric emptying is modulated not only by the nervous system and brain-gut peptides, but also involves coordinated interactions among GSMCs, ICCs, and various immune cell populations (19). GSMCs are key components of the gastric wall that mediate the rhythmic contractions and relaxations of the stomach. Since reduced propulsive contractions in the gastric antrum can impair the grinding, mixing, and expulsion of food into the duodenum, the proper function of these specialized cells is crucial for proper gastric emptying (54). Numerous studies have shown that prolonged exposure to hyperglycemic conditions induces apoptosis in GSMCs, resulting in significant alterations to gastric smooth muscle morphology and contractile function, which collectively contribute to the pathogenesis of DGP (55–58). Therefore, attenuating GSMCs damage may represent an important therapeutic target for the treatment of diabetic gastroparesis.

**Figure 3:**
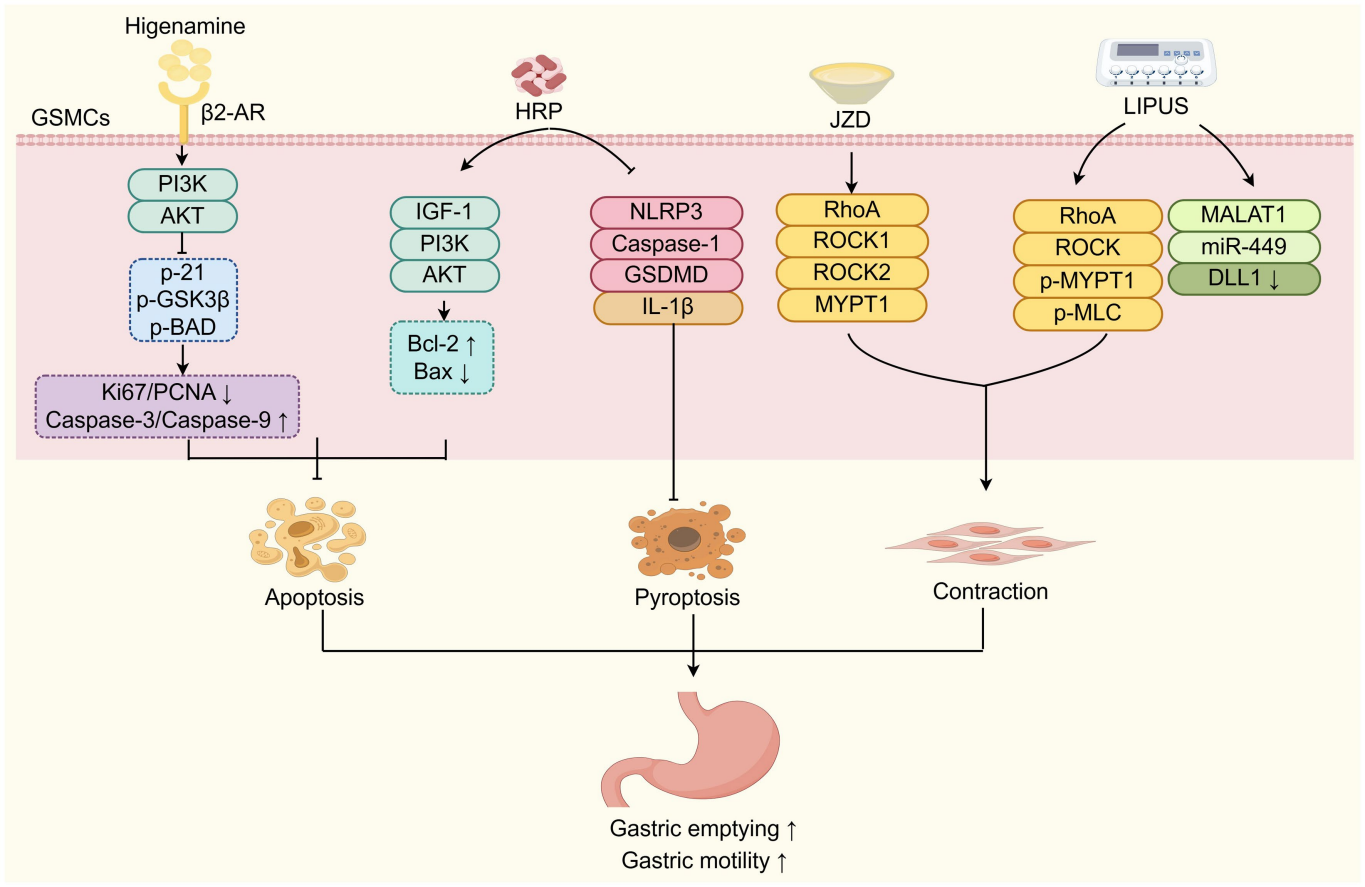
TCM improves DGP by attenuating GSMCs damage. TCM components (HRP, Higenamine, JZD) and LIPUS protect GSMCs from damage (apoptosis, pyroptosis) and enhance contraction via diverse signaling pathways to improve DGP. HRP: Hedysari Radix Polysaccharide; JZD: Jinqi Zhizhu Decoction; LIPUS: low-intensity pulsed ultrasound stimulation; GSMCs: gastric smooth muscle cells.

Emerging evidence has shown that TCM improves DGP by attenuating GSMCs damage (Figure 3). Research has shown that Hedysari Radix Polysaccharide (HRP), isolated from dried roots of the classic TCM herb *Hedysarum polybotrys* Hand-Mazz. (Hong qi), can help protect gastric antral SMCs from apoptosis and promote gastric smooth muscle contraction by activating the IGF-1/PI3K/AKT signalling pathway, thereby alleviating DGP (59). Additionally, HRP has been displayed to decrease the mRNA and protein expressions of NLRP3, Caspase-1, GSDMD, and IL-1β in gastric antrum tissue, and thus inhibit the pyroptosis of GSMCs and promote the gastric emptying (60). Pyroptosis is a caspase-dependent, inflammatory programmed cell death mediated by Gasdermin proteins. The inflammatory cascade triggered by the persistent high glucose and high-fat conditions of diabetes may lead to the pyroptosis of GSMCs in the gastric antrum, ultimately resulting in decreased gastric motility and impaired gastric emptying (61). Higenamine, a plant alkaloid first isolated in 1976 from the TCM herb *Aconitum carmichaelii* Debeaux. (fuzi) root and the main active ingredient in Fuzi Lizhong Decoction, has been shown to inhibit the apoptosis of GSMCs in DGP rats by activating the β2-AR/PI3K/AKT pathway (62). Furthermore, Wan et al. (49) demonstrated that Jinqi Zhizhu Decoction (JZD) effectively restored ultrastructural integrity in GSMCs of DGP rats, ameliorating multiple pathological alterations, including: (i) disrupted intercellular junctions, (ii) basement membrane fragmentation, (iii) organelle depletion, (iv) mitochondrial degeneration, and (v) cytoplasmic vacuolization in the gastric antral region. It also promoted the contraction of gastric smooth muscle by upregulating the RhoA/ROCK (Ras homolog family member A/Rho-associated kinase) signalling pathway in gastric tissue (48). In addition, low-intensity pulsed ultrasound stimulation (LIPUS) at acupoint ST36 (Zusanli) enhances the contractile ability of GSMCs in DGP rats by regulating the RhoA/ROCK and MALAT1/miR-449a/DLL1 signalling pathways, ultimately improving the gastric emptying (63). Studies have shown that in gastric smooth muscle tissue, activated RhoA can enhance MLC20 phosphorylation by binding to the MYPT1 subunit of its downstream effector molecule ROCK and Myosin Light Chain Phosphatase (MLCP), thereby promoting gastric smooth muscle contraction (64). Moreover, mechanistic studies have revealed that MALAT1 can promote phenotypic switching of GSMCs under high-glucose conditions through the miR-449a/DLL1 axis, thereby maintaining cell survival (65). In summary, TCM approaches such as oral administration of Hedysari Radix Polysaccharide, higenamine, Jinqi Zhizhu Decoction, and LIPUS, can protect GSMCs from apoptosis, pyroptosis, and other forms of injury by targeting multiple signalling pathways, showing therapeutic potential in improving gastrointestinal motility of DGP.

Nevertheless, current research on TCM-mediated GSMCs protection in DGP is constrained by small sample sizes and lack of blinding, with bias risks arising from inconsistent outcome measures, Future studies should focus on standardized protocols and multi-center blinded trials to enhance evidence reliability.

### 2.4 Regulating inflammation and immunity

There is accumulating evidence that demonstrates a significant association between elevated systemic inflammatory cytokines, such as IL-6, IL-8, IL-10, IFN-*γ*, and TNF-*α* and impaired gastrointestinal motility (66–68). Mechanistic studies reveal that pro-inflammatory cytokines, particularly TNF-α, IL-1β, and IL-6, contribute to hyperglycemia-induced enteric neuronal apoptosis and disruption of the ICC network both structurally and functionally (67, 68). In contrast, IL-10 has been demonstrated to enhance gastric emptying in diabetic mice by upregulating the anti-inflammatory cytokine heme oxygenase 1 (HO-1) levels and restoration of ICC network integrity (69). Reduced IL-10 levels may exacerbate the detrimental effects of pro-inflammatory cytokines on intestinal mucosa, promoting localized inflammation, cytotoxic responses, and subsequent immune dysregulation (70). Clinical trials and experimental studies further support these findings. Gastric biopsies from both DGP patients and animal models consistently show a decrease in anti-inflammatory macrophages, along with upregulated pro-inflammatory macrophage-associated gene expression. This suggests that macrophage-mediated immune imbalance and inflammatory responses play a crucial role in the development of delayed gastric emptying in diabetes (71, 72). Given these pathophysiological mechanisms, TCM interventions may present a promising therapeutic strategy for DGP.

**Figure 4:**
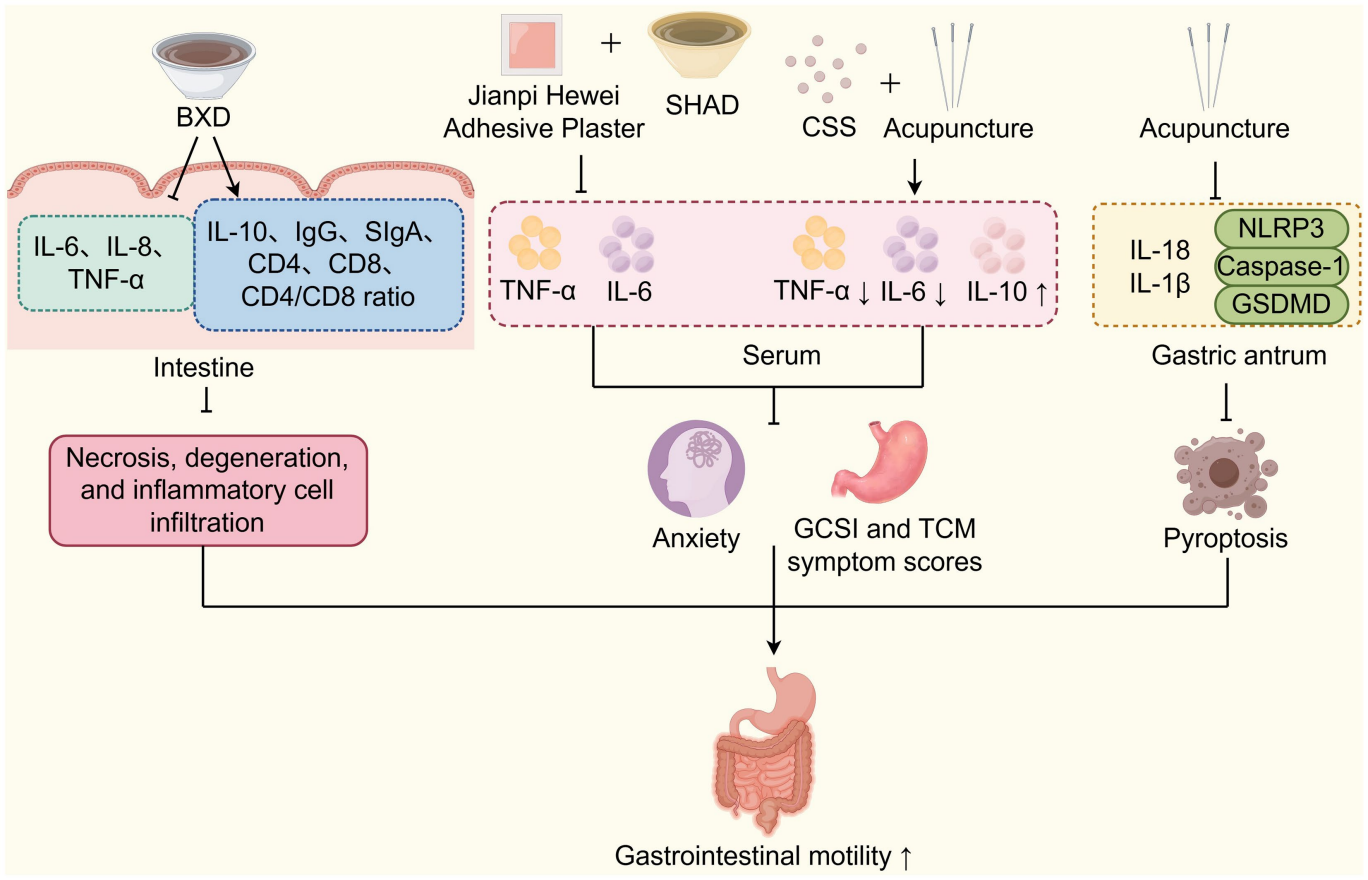
TCM improves DGP by regulating inflammation and immunity. TCM interventions (BXD, Jianpi Hewei Adhesive Plaster, SHAD, CSS, and acupuncture) regulate inflammation, immunity, and pyroptosis via cytokine modulation to improve DGP-related gastrointestinal motility. SHAD: Sini Hewei Anshen Decoction; CSS: Chaihu Shugan San.

Multiple studies have demonstrated that TCM improves DGP by regulating inflammation and immunity (Figure 4). Yang et al. (73) study found that Banxia Xiexin Decoction can reduce the levels of inflammatory factors such as IL-6, IL-8, and TNF-*α*. It also upregulates the levels of the anti-inflammatory factor IL-10, as well as immune-related indicators like IgG, SIgA, CD4, CD8, and the CD4/CD8 ratio in the intestinal mucosa. CD8 molecules are mainly expressed in cytotoxic T lymphocytes (CTLs) and play an essential role in cellular immunity, while CD4 molecules assist B cells in antibody production and can also induce inhibition of T cell functions by secreting cytokines. The CD4/CD8 ratio serves as a key indicator of a stable immune environment in the body, with any increase or decrease reflecting changes in immune function (73). Immunoglobulin G (IgG) is the primary protective immunoglobulin in the human body, preventing the invasion of foreign pathogenic microorganisms and antigens. In contrast, SIgA is the major immunoglobulin in the intestinal mucosa that defends against the invasion of commensal bacteria and pathogens, playing a crucial role in maintaining intestinal mucosal homeostasis. A local depletion or reduction of SIgA can lead to recurrent intestinal infections, which stimulate pro-inflammatory cytokines including IL-6, IL-8, and TNF-*α*, ultimately impairing gastrointestinal motility and gastric emptying (74, 75). These effects reverse pathological changes in the intestinal mucosal muscle layer of DGP rats, including atrophy, necrosis, submucosal degeneration, and inflammatory cell infiltration, thereby improving gastrointestinal motility and gastric emptying (73). Huang et al. (76) research demonstrated that the Spleen-Strengthening and Stomach-Regulating Plaster for umbilical application combined with the oral administration of the modified Sini Hewei Anshen Decoction (SHAD). SHAD includes the TCM herbs such as *Bupleurum chinense* DC. (Chaihu), *Paeonia lactiflora* Pall. (Baishao), *Citrus aurantium* L. (Zhike), *Atractylodes macrocephala* Koidz. (Baizhu), *Citrus reticulata* Blanco (Chenpi), *Aucklandia costus* Falc. (Muxiang), *Panax ginseng* C. A. Mey. (Renshen), *Dioscorea opposita* Thunb. (Shanyao), *Polygala tenuifolia* Willd. (Yuanzhi), *Acorus tatarinowii* Schott. (Shichangpu), and *Glycyrrhiza uralensis* Fisch. ex DC. (Gancao). This combined intervention effectively improved the Gastrointestinal Clinical Symptom Index (GCSI) score and gastric emptying time in patients with DGP associated by anxiety due to liver-stomach disharmony. These improvements were achieved by reducing the serum levels of inflammatory factors such as TNF-*α* and IL-6 (76). Chaihu Shugan San, a TCM decoction consisting of *Paeonia lactiflora* Pall. (Baishao), *Bupleurum chinense* DC. (Chaihu), *Citrus aurantium* L. (Zhike), *Cyperus rotundus* L. (Xiangfu), *Citrus reticulata* Blanco. (Chenpi), *Ligusticum chuanxiong* Hort. (Chuanxiong), and *Glycyrrhiza uralensis* Fisch. ex-DC. (Gancao), is a classical prescription to promote Qi and blood circulation. Guo et al. (77) have discovered that Chaihu Shugan San decoction in combination with acupuncture at specific acupoints (Zusanli and Guanyuan as main acupoints, Zhongwan and Fenglong as auxiliary acupoints), can invigorate the spleen and stomach, and soothe the liver and relieve depression. Mechanism exploration has found that this combined TCM intervention can reduce serum TNF-*α* and IL-6 levels, while increasing IL-10 levels to increase gastric motility and improves the clinical symptoms of DGP patients. Similarly, Xiao et al. (78) discovered that electroacupuncture at Zusanli, Liangmen, and Sanyinjiao acupoints could significantly reduce the levels of IL-18, IL-1β and the NLRP3 inflammasome in gastric antrum tissue, leading to a significant increase in gastric emptying rate and small intestinal propulsion rate in DGP rats. Additionally, electroacupuncture at these acupoints could repair the pathological damage in the gastric antrum tissues and improve gastric emptying. Mechansitically, this intervention has been found to inhibit NLRP3/caspase-1/GSDMD pathway to block pyroptosis in DGP murine model (79). In summary, these studies demonstrate that TCM approaches, such as Banxia Xiexin Decoction, Sini Hewei Anshen Decoction, Chaihu Shugan San, Spleen-Strengthening and Stomach-Regulating Plaster for umbilical application, and electroacupuncture at specific acupoints, can effectively reduce inflammatory factors, modulate immune responses, enhance gastrointestinal motility, and thereby prevent and treat DGP.

However, most of these studies have small sample sizes and lack adequate blinding, leading to potential bias. This is partly because of the difficulty in blinding umbilical plaster and acupuncture interventions, and future work should explore innovative blinding strategies alongside larger cohort studies.

### 2.5 Inhibiting oxidative stress

Oxidative stress refers to an imbalance between the production of oxygen free radicals, such as reactive oxygen species (ROS) and reactive nitrogen species (RNS), and the body’s antioxidant defense system, leading to tissue damage. While contributing to the development of various complications of diabetes, oxidative stress also triggers gastric mucosal damage and gastric motility disorders, playing a significant role in the onset and progression of DGP (54, 80, 81). Impaired formation of the ICCs network and extracellular matrix remodeling of GSCMs due to oxidative stress have been identified as key causes of DGP (54, 82). In DGP rats, gastric motility is impaired, with increased levels of malondialdehyde (MDA), a marker of oxidative stress in gastric tissue, and decreased levels of serum superoxide dismutase (SOD), an antioxidant enzyme (83). Additionally, a decline in HO-1-positive macrophages has been reported in rat models of DGP, suggesting that increased oxidative stress and failure of the antioxidant response may play an important role in the pathogenesis of DGP (84).

**Figure 5:**
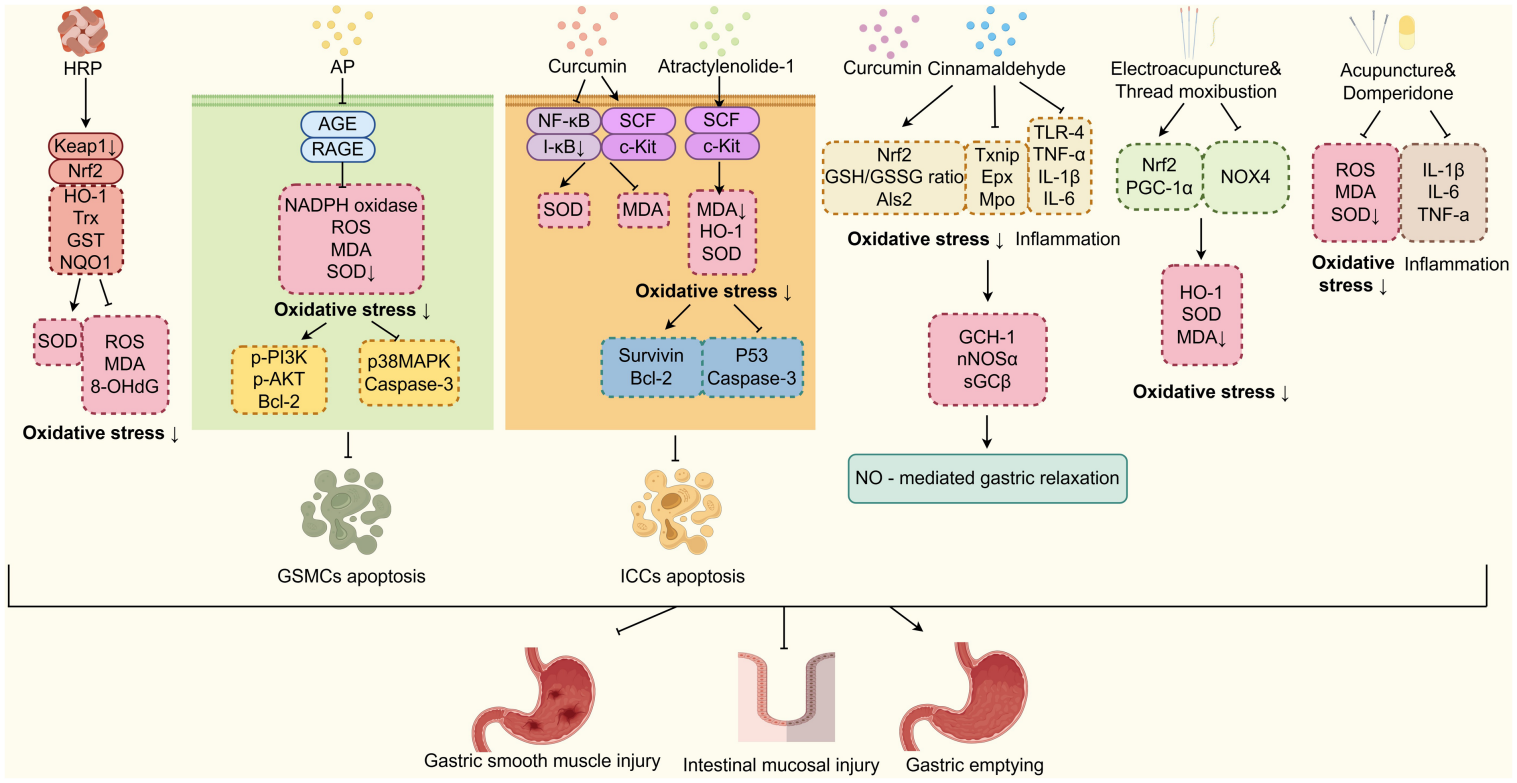
TCM improves DGP by inhibiting oxidative stress. TCM-based interventions (HRP, AP, curcumin, etc.) mitigate DGP. They inhibit oxidative stress via pathways, such as Nrf2, AGE/RAGE, and NF-κB, to reduce apoptosis of GSMCs/ICCs and improve gastric function. HRP, Hedysari Radix Polysaccharide; AP, *Alpinia officinarum* Hance-*P. cablin* (Blanco) Benth; Nrf2, Nuclear factor erythroid 2-related factor 2.

Through the inhibition of oxidative stress, multiple kinds of TCM compounds can attenuate diabetic gastroparesis (Figure 5). Guo et al. (85) have found that *Hedysari Radix* Polysaccharide (HRP) can reduce the levels of Kelch-like ECH-associated protein 1 (Keap1) mRNA while increasing the levels of Nuclear factor erythroid 2-related factor 2 (Nrf2), HO-1, thioredoxin (Trx) mRNA, glutathione S-transferase (GST), and quinone oxidoreductase 1 (NQO1) proteins. Consequently, HRP reduces the levels of ROS, MDA and 8-hydroxydeoxyguanosine (8-OHdG), while enhancing the levels of SOD in DGP rats. These findings have suggested that HRP plays a crucial role in DGP therapeutics by inhibiting oxidative stress, repairing small intestinal mucosal damage, and improving gastric emptying and small intestinal propulsion rates. *Alpinia officinarum* Hance (Gaoliangjiang) and *P. cablin* (Blanco) Benth (Guanghuoxiang) are TCM plants that have been used as natural remedies for managing diabetes and gastrointestinal issues. According to TCM theory, their therapeutic mechanisms involve the regulation of spleen-stomach meridians and promoting gastric motility. A recent study has demonstrated that *A. officinarum* Hance-*P. cablin* (Blanco) Benth (AP) drug pair can inhibit the GSMCs apoptosis by reducing oxidative stress levels through downregulating the AGE/RAGE axis, thereby increasing the gastric emptying rate (86). Curcumin, the main active ingredient of turmeric isolated from the TCM plant *Curcuma Longa* L. (Jianghuang), has been shown to inhibit the ICCs apoptosis and improve gastric emptying through attenuating oxidative stress and nuclear factor kappa B (NF-κB) activation in gastric tissues of DGP rats (87). Furthermore, recent studies have demonstrated that antioxidant compounds, such as curcumin and cinnamaldehyde, an active constituent derived from the TCM herb *Cinnamomum cassia* Presl. (Rougui), can ameliorate inflammation and oxidative stress associated with obesity-induced chronic diabetes. This therapeutic effect subsequently restores nitrergic (NO-mediated) neural regulation of gastric motility and emptying function. The underlying mechanisms involve: (i) upregulation of Nrf2 expression and GSH/GSSG ratio, regulating oxidative stress marker genes expression (Txnip, Als2, Epx, and Mpo); (ii) downregulation of Toll-like receptor 4 (TLR4) signalling and suppression of pro-inflammatory cytokines (TNF-*α*, IL-1β, IL-6); (iii) restoration of nNOS cofactor BH4 synthesis via GCH-1 upregulation; and (iv) enhancement of nNOSα protein expression, dimerization, and soluble guanylate cyclase (sGC) activity. These effects were observed in wild-type mice, but not in Nrf2 knockout (Nrf2 KO) mice (88). The study conducted by Hui et al. (1) revealed that atractylenolide-1, one of the primary bioactive compounds of TCM herb *Atractylodes macrocephala* Koidz (Baizhu), can regulate oxidative stress responses and protect ICCs from apoptosis, and restore the gastric tissue network structure in the DGP rat model by activating the SCF/c-kit signalling pathway. Moreover, electroacupuncture combined with “Zhuang” medicinal thread moxibustion, a unique moxibustion practice of the Zhuang ethnic group in China, was found to inhibit oxidative stress-induced damage to DGP rat model. This was achieved through the upregulation of expression of HO-1, Nrf2, PGC-1*α* proteins and mRNA, and promotion of Nrf2 nuclear translocation in gastric antrum tissue (89). Acupuncture targeting the Baihui (GV20), Shenting (GV24), Zhongwan (CV12), Zusanli (ST36), Hegu (LI14), and Taichong (LR3) acupoints, in combination with oral use of domperidone, has been shown to reduce the serum levels of ROS, MDA, TNF-α, IL-6 and IL-1β levels, while increasing the SOD activity, thereby inhibiting oxidative stress and inflammatory responses. As a result, it alleviates clinical symptoms and improves the gastric emptying rate in DGP patients diagnosed with a liver stagnation and spleen deficiency pattern (90). These findings suggest that TCM approaches, including the oral administration of Hedysari Radix Polysaccharide, curcumin, cinnamaldehyde, oral or external use of *A. officinarum* Hance-*P. cablin* (Blanco) Benth drug pair, as well as electroacupuncture and moxibustion, can effectively treat DGP by reducing oxidative stress levels, decreasing inflammation, and mitigating damage to ICCs, GSMCs, and gastric smooth muscle.

Notably, current studies on TCM inhibiting oxidative stress in DGP are limited by small samples and lack of blinding, with bias risks from variable oxidative stress marker detection methods. Future research should adopt standardized assays and large-scale blinded RCTs to strengthen evidence.

### 2.6 Regulating gut microbiota imbalance

The gut microbiota refers to the microorganisms present in the human digestive system, including up to 1,000 species of bacteria and trillions of individual microorganisms. Under normal circumstances, the gut microbiota plays important roles in digestion and absorption, immune protection, and nutrient synthesis (91). It is closely involved in the absorption, secretion, and metabolism of the digestive tract. When the balance of the gut microbiota is disrupted, it can lead to the development of various diseases. Studies have found a strong link between diabetes and the gut microbiota (92, 93). Individuals with diabetes often exhibit decreased levels of beneficial probiotics such as *Lactobacillus* and *Bifidobacterium,* while pathogenic and potentially harmful bacteria, including Gram-negative bacteria and *Enterobacter*, become more abundant. This microbial imbalance can disrupt intestinal barrier function, increase mucosal permeability and trigger systemic inflammation, leading to increased endotoxin production and gastrointestinal dysfunction, contributing to the development of DGP (92–94). In addition, the gut microbiota, including *Bifidobacterium*, *Bacteroides*, and *Prevotella*, can break down polysaccharides that are difficult for the human body to absorb, generating short-chain fatty acids. These short-chain fatty acids can activate G protein-coupled receptors 41 and 43, promoting the secretion of 5-HT, which works in coordination with the ENS, muscles, and the network of ICCs to regulate the gastrointestinal secretion and intestinal motility (39, 74, 95). Dysbiosis of the gut microbiota can also affect the secretion of GLP-1, bile acid metabolism, vitamin B12 synthesis, TLR4/NF-κB signalling pathway, thereby exacerbating insulin resistance, glucose tolerance abnormalities, intestinal inflammatory response, and oxidative stress, ultimately leading to delayed gastric emptying (74). Therefore, regulating the gut microbiota imbalance may be a novel target for the treatment of DGP.

**Figure 6:**
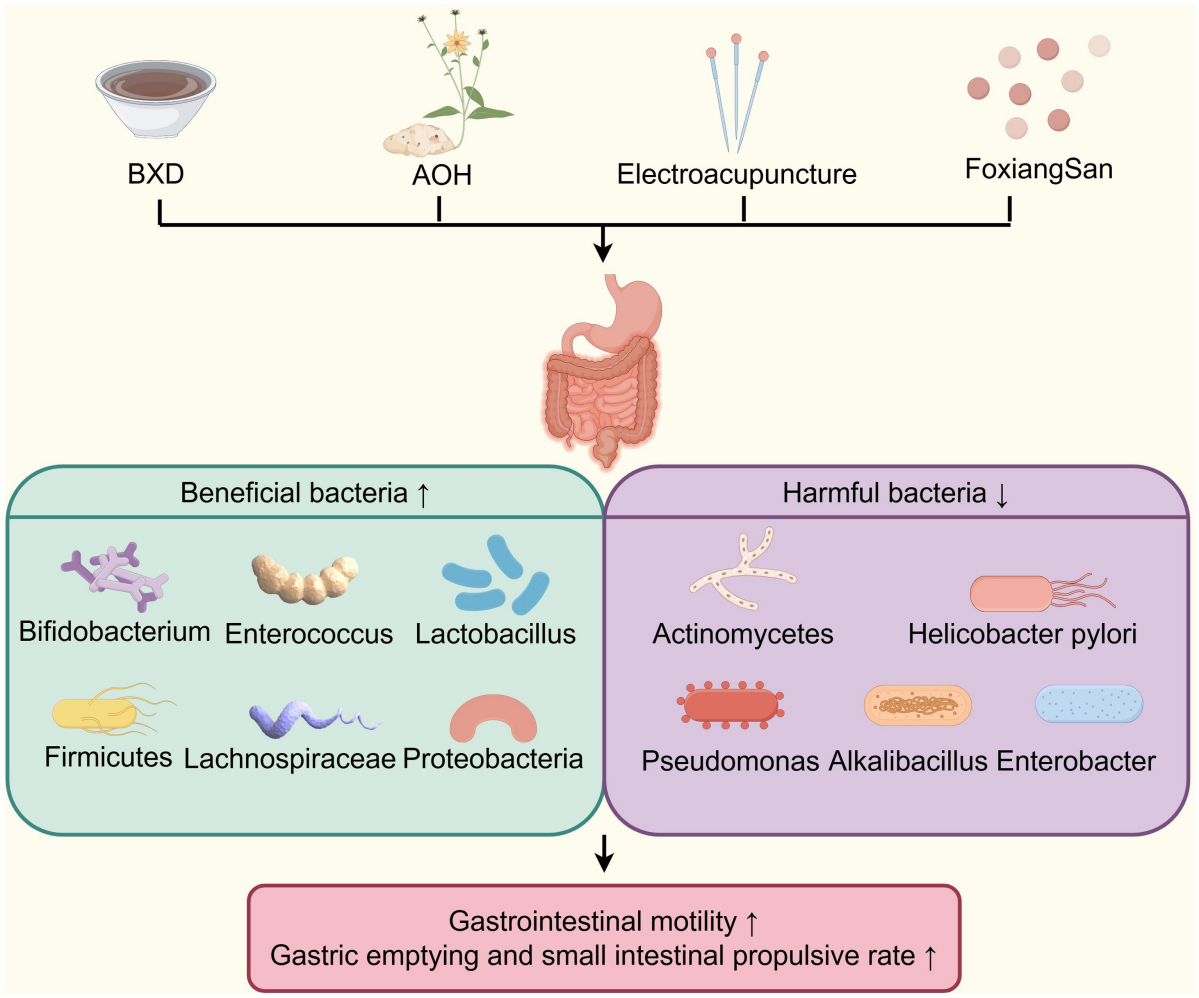
TCM improves DGP by restoration of gut microbiota homeostasis. TCM (BXD, AOH, Electroacupuncture and FoxiangSan) improves DGP by restoring gut microbiota homeostasis, enriching beneficial bacteria (e.g., Bifidobacterium) and depleting harmful ones (e.g., *Helicobacter pylori*) to enhance gastrointestinal motility. AOH, *Alpinia officinarum* Hance.

TCM improves DGP by restoration of gut microbiota homeostasis (Figure 6). For example, Xu et al. (73, 96) discovered that in DGP murine model, gut microbiota imbalance elevated pro-inflammatory cytokines, disruption of the intestinal mucosal immune barrier, and gastrointestinal motility dysfunction. BXD was found to reverse these conditions by increasing the abundance of beneficial bacteria such as *Bacteroides*, *Bifidobacterium*, *Enterococcus*, and *Lactobacillus*, while reducing the number of pathogenic bacteria *Enterobacter.* Additionally, as mentioned above, BXD enhances serum levels of IgA, IL-10, CD4, CD8, and CD4/CD8 ratio levels, while reducing serum endotoxin, intestinal IgG, IL-6, IL-8, and TNF-*α* levels. These findings suggest that BXD improves gastrointestinal motility through multiple mechanisms, including alleviating intestinal tissue inflammation, repairing the mucosal immune barrier, and regulating gut microbiota imbalance. *Alpinia officinarum* Hance (AOH), a plant from the Zingiberoside family rich in diarylheptanoids and flavonoids, has been widely used in TCM for thousands of years. Research has shown that it can significantly accelerate gastrointestinal propulsion rate in DGP rats by rebalancing the gut microbiota, such as increasing the proportion of *Firmicutes* and the relative abundance of *Proteobacteria and Lachnospiraceae*_NK4A136_group, reducing the relative abundance of *Bacteroidota* (97). FoxiangSan, formulated by National TCM Master Professor Lv Renhe, comprises *Citrus medica* L.var.socdactylis Swingle (Foshou), *Citrus medica* L. (Xiangyuan), *Cyperus rotundus* L. (Xiangfu), *Citrus Reticulatae Pericarpium* (Chenpi), *Inula japonica* Thunb. (Xuanfuhua), *Lindera aggregata* (Sims) Kosterm. (Wuyao), and *Pseudostellaria heterophylla* (Miq.) Pax ex Pax et Hoffm. (Taizishen), has been demonstrated to improve gastric emptying in DGP rats by decreasing the abundance of *Bacteroidetes*, *Actinobacteria*, and *Helicobacter pylori,* increasing the abundance of *Firmicutes* and *Lachnospiraceae* (98). Research has shown that *Actinobacteria* play an essential role in maintaining the balance of gut microbiota (99, 100). Furthermore, *Helicobacter pylori* infection was found in 74.6% of patients with DGP, significantly higher than in the diabetic-only and normal control groups (101). Thus, FoxiangSan may enhance gastric emptying in diabetes by regulating the levels of *Helicobacter pylori* and *Actinobacteria*. Electroacupuncture on acupoints such as Zhongwan (CV12), Tianshu (ST25), Zhongfu (LU1), bilateral Zhangmen (LR13), bilateral Taibai (SP3), bilateral Taiyuan (LU9), bilateral Zusanli (ST36), and bilateral Shangjuxu (ST37), based on the TCM theory of “ascending lucidity and descending turbidity,” were found to upregulate the relative abundance of beneficial bacteria (*Proteobacteria, Olsenella* and *Christensenellaceae R-7*), and inhibit the relative abundance of pathogenic bacteria,(*Pseudomonas* and *Alkalibacterium*) in patients with diabetic gastrointestinal dysfunction (102). *Olsenella*, a genus of Gram-positive, anaerobic, non-spore-forming bacteria belonging to the family *Atopobiaceae* (*phylum Actinobacteria*), is closely associated with gastrointestinal immune function (103). *Christensenellaceae R-7* is a strain within the family *Christensenellaceae* (*phylum Firmicutes*). As a probiotic factor, it shows a significant negative correlation with inflammatory reactions and metabolic diseases (104). Therefore, the “increasing clearance and reducing turbidity” approach may enhance the gastrointestinal inflammatory immune response by boosting the abundance of certain probiotics, thus alleviating gastrointestinal dysfunction in diabetic patients. In conclusion, these studies suggest that TCM, such as Banxia Xiexin decoction, FoxiangSan, and electroacupuncture can effectively treat DGP by promoting the abundance of beneficial gut microbiota and suppressing harmful microbiota. It should be noted that BDX, FoxiangSan, and AOH exert distinct regulatory effects on the relative abundance of *Bacteroidota,* with BDX upregulating its abundance while FoxiangSan and AOH downregulate it. *Bacteroidota* plays a critical role in modulating gastric emptying through multiple mechanisms, including impacting short-chain fatty acid production, bile acid metabolism, and gut immune function (105–107). Previous studies have demonstrated variable effects of *Bacteroidota* on either promoting or delaying gastric emptying, which may be attributed to differences in microbial composition and host microenvironmental factors (107, 108). Consequently, future research should integrate metagenomic analyses with functional validation studies, including bacterial strain colonization experiments, to further elucidate the precise mechanisms through which TCM regulate the gut microbiota for the treatment of DGP.

Additionally, most existing studies on TCM regulating gut microbiota in DGP have small sample sizes and lack blinding, introducing potential bias. This is due in part to the high cost of metagenomic analyses in large cohorts, and future work should balance depth of microbial analysis with larger sample sizes to improve result generalizability.

## 3 Conclusion and perspective

DGP, a common manifestation of diabetic autonomic neuropathy, is characterized by impaired gastric motility and delayed gastric emptying. Affected patients frequently present with symptoms such as postprandial fullness, early satiety, chronic nausea, and other gastrointestinal disturbances, all of which substantially diminish their quality of life. Current therapeutic approaches, including prokinetic medications and surgical interventions, primarily focus on restoring gastric motility and managing symptoms, with limited attention to the disease’s underlying pathogenesis. Their efficacy remains suboptimal, often accompanied by adverse effects and high recurrence rates in certain patient populations. Therefore, there is an urgent need for treatment alternatives that are more targeted to the underlying mechanisms of DGP, and that are safer, more effective, and sustainable. In this review, based on the available evidence and the latest research, we comprehensively evaluated the impact of TCM in both DGP patients and model animals, as well as the possible mechanisms involved. It has been demonstrated that TCM holds considerable promise in treating DGP, with multifaceted strategies including the oral administration of TCM decoctions, active ingredients from medicinal herbs, and external therapies including acupuncture and moxibustion. These approaches target multiple mechanisms, including the regulation of ICCs and GSMCs injury, brain-gut peptide dysregulation and neuropathy, inflammatory and immune responses, oxidative stress, and gut microbiota imbalance.

Despite these advantages, this review still has several limitations. Firstly, relatively few clinical trials explore the mechanisms of TCM in treating DGP, featuring small sample sizes and predominantly Chinese study populations. This imbalance between robust basic research and weak clinical evidence specifically restricts the clinical promotion of TCM for DGP: it fails to meet international evidence-based medicine standards, limits applicability across diverse ethnic groups, and impedes inclusion in global clinical practice guidelines. To bridge this gap, future studies should prioritize multicenter, large-sample randomized controlled trials, enroll multi-ethnic populations to improve generalizability, integrate syndrome differentiation into study designs for stratified analysis, and conduct long-term follow-ups to verify long-term efficacy and safety.

Secondly, while multiple studies have confirmed that TCM alleviates DGP through diverse mechanisms. For instance, it has been found that Banxia Xiexin Decoction modulates ICCs proliferation, gut microbiota, inflammatory responses, and immune dysregulation simultaneously. Howerver, key gaps persist, particularly regarding the bidirectional regulation of specific gut microbial genera (e.g., Bacteroidota) by different TCMs: Banxia Xiexin Decoction upregulates Bacteroidota abundance, whereas FoxiangSan and *Alpinia officinarum* Hance downregulate it. This regulatory difference may stem from the functional heterogeneity of Bacteroidota, as a high-impact study revealed that Bacteroidales, the dominant order in Bacteroidota, exhibit strain-specific fitness variations driven by differences in Acyl-CoA thioesterase activity and nucleotide polymorphisms regulating Acyl-CoA transferase, which further determine their sensitivity to metabolites including butyrate and ability to adapt to glycan environments. While we hypothesize that the multi-target effects of TCM, the theory has not yet been confirmed by direct experimental evidence. To clarify the specific role of Bacteroidota in TCM-mediated DGP relief will require functional validation experiments such as strain-level colonization in gnotobiotic mice and metabolomic analysis of Bacteroidota-derived metabolites.

Additionally, it has been demonstrated that the PI3K/AKT pathway exert opposing roles in the TCM treatment of DGP. To be specific, electroacupuncture ameliorates DGP by downregulating the PI3K/AKT/mTOR pathway to enhance ICCs autophagy, while Hedysari Radix Polysaccharide, Higenamine, and the *A. officinarum*–*P. cablin* drug pair treat DGP by activating the IGF and β2-AR to upregulate p-PI3K/p-AKT expression, inhibiting GSMCs apoptosis. The opposing regulation of PI3K/AKT in DGP may stem from its multifaceted roles in distinct pathophysiological processes, particularly driven by cell type heterogeneity and spatial compartmentalization of signaling molecules. Single-cell sequencing of gastric muscle tissue from gastroparesis patients reveals that muscularis macrophages (MM) exhibit altered expression of tissue-protective and stromal-activating genes, remodeling the microenvironment of ICCs and GSMCs and modulating their responsiveness to PI3K/AKT regulation. Moreover, AKT activation is strictly compartment-specific: electroacupuncture inhibits plasma membrane-localized AKT in ICCs to suppress mTORC1 and promote autophagy, while TCM components activate nuclear or endosomal AKT in GSMCs to regulate apoptotic transcription factors like FOXO1. This spatial-dependent pattern aligns with findings in gastric adenocarcinoma, where spatial transcriptomics shows cell microenvironment (e.g., tumor-stroma interface) dictates PI3K/AKT activity, supporting that cell localization and microenvironment together shape the pathway’s function in DGP. For example, PI3K/AKT/mTOR activation typically inhibits autophagy but suppresses apoptosis, reflecting dual regulatory effects, and TCM’s multi-target, multi-pathway properties also help explain this differentiation. However, it should be noted that further in-depth research is needed to clarify the exact role of PI3K/AKT in DGP and its potential regulatory network, as current hypothesis relies on indirect evidence from spatial transcriptomics and pathway correlation analyses rather than direct functional validation.

To further address these limitations and promote the application of TCM in the treatment of DGP and expand its global acceptance in evidence-based medicine, further efforts are warrant. In the context of current Western treatments for DGP, prokinetics (e.g., domperidone) often relieve symptoms but carry risks of extrapyramidal effects, gastric electrical stimulation (GES) is invasive and effective only in select patients, and surgery is reserved for severe, refractory cases. To tackle these drawbacks, TCM can serve as a complementary or alternative option, offering advantages of multi-target mechanism regulation, favorable safety profiles, and ability to address both symptoms and underlying pathogenesis, especially for patients intolerant to Western drugs or ineligible for invasive interventions. Among TCM’s multiple therapeutic mechanisms for DGP, ICCs protection may act as the core driver for improving gastric emptying, given ICCs’ essential role as gastric motility pacemaker cells, without intact ICCs function, other regulatory effects, such as the brain-gut peptide modulation, struggle to restore normal gastric motility. Gut microbiota regulation, by contrast, exerts a synergistic effect: it enhances ICCs protection indirectly via metabolites like short-chain fatty acids that support ICCs survival and function. Future studies can use ICCs knockout models or gut microbiota depletion approaches to verify this priority and synergistic relationship.

Furthermore, more focus should be put on conducting clinical randomized controlled trials that adhere to the principles of syndrome differentiation and treatment, while exploring the mechanisms of TCM from multiple dimensions. For complex Chinese herbal compounds like Banxia Xiexin Decoction, modern technologies can be leveraged to screen key active components: network pharmacology can construct “component-target-disease” interaction networks to predict core ingredients targeting DGP-related pathways (PI3K/AKT and inflammatory signaling,etc.), while metabolomics can track *in vivo* absorption, distribution, metabolism, and excretion of compounds to identify bioavailable active metabolites. Additionally, the synergistic mechanism of components, especially scientific validation of the “monarch-minister-adjuvant-courier” theory, requires further research. The future research may include the component knockout/addition experiments in DGP models to verify whether monarch herbs, (e.g., Pinelliae Rhizoma in Banxia Xiexin Decoction) play a core role and minister herbs (e.g., Scutellariae Radix), enhance efficacy or reduce toxicity.

In addition, by using modern techniques such as *in situ* hybridization, immunohistochemistry, and gene chips, we can explore the mechanism of action of natural products from TCM and study the direct targets of these candidate drugs. Alternatively, we can rely on modern analytical methods and translational models, such as using network pharmacology to analyze the potential targets of natural products from TCM. Moreover, it is essential to utilize high-throughput 16S rRNA gene sequencing and metagenomics to comprehensively characterize how TCM regulate the composition, diversity, and functional potential of the gut microbiota in patients with DGP, and observe whether specific beneficial bacterial genera are enriched or harmful bacterial genera are inhibited. At the same time, metabolomics should be employed to analyze serum, urine, and fecal samples to identify key metabolite changes induced by TCM treatment, such as short-chain fatty acids, tryptophan metabolites, bile acids, neurotransmitters and their precursors. This multi-omics integration approach is indispensable for mapping complex interaction networks. These will provide a necessary pre-clinical basis for the wide application of natural products from traditional Chinese medicine. Additionally, in TCM research on gastroparesis, organoids can mimic gastric tissue properties, and single-cell omics can analyze cellular heterogeneity; their combination facilitates mechanism elucidation. These efforts with novel research techniques will lay a more solid scientific foundation for the development and application of TCM as a complementary or alternative approach to conventional treatments in the prevention and treatment of DGP, ultimately providing patients, particularly those with limited efficacy or adverse effects from previous treatments, with safer and more effective therapeutic options.
